# Effect of larval diets on the life table parameters of dengue mosquito, *Aedes aegypti* (L.) (Diptera: Culicidae) using age-stage two sex life table theory

**DOI:** 10.1038/s41598-023-39270-8

**Published:** 2023-07-24

**Authors:** Muhammad Salim, Muhammad Kamran, Inamullah Khan, Ahmad Ur Rahman Saljoqi, Sarir Ahmad, Mikhlid H. Almutairi, Amany A. Sayed, Lotfi Aleya, Mohamed M. Abdel-Daim, Muddaser Shah

**Affiliations:** 1 Department of Plant Protection, Faculty of Crop Protection Sciences, The University of Agriculture, Peshawar, Pakistan; 2 Pakistan Atomic Energy Commission, Nuclear Institute for Food and Agriculture (NIFA), P. O. Box 446, Peshawar, Pakistan; 3 Department of Entomology, Abdul Wali Khan University Mardan, Mardan, 23200 Pakistan; 4 Department of Zoology, College of Science, King Saud University, P.O. Box 2455, 11451 Riyadh, Saudi Arabia; 5 Zoology Department, Faculty of Science, Cairo University, Giza, 12613 Egypt; 6 Chrono-Environnement Laboratory, UMR CNRS 6249, CEDEX, Bourgogne, Franche-Comté University, 25030 Besançon, France; 7 Pharmacology Department, Faculty of Veterinary Medicine, Suez Canal University, Ismailia, 41522 Egypt; 8 Natural and Medical Sciences Research Center, University of Nizwa, Birkat Al Mauz, P.O. Box 33, Nizwa, 616 Oman; 9 Department of Botany, Abdul Wali Khan University Mardan, Mardan, 23200 Pakistan

**Keywords:** Biotechnology, Genetics, Zoology, Climate sciences, Environmental sciences

## Abstract

The current study regarding the effects of larval diets on the life table parameters of dengue mosquitoes, *Aedes aegypti* was conducted under laboratory conditions at 27 ± 2 °C and 60 ± 5% relative humidity at NIFA (Nuclear Institute for Food and Agriculture) Peshawar, Pakistan. The data on life table parameters of *Ae. aegypti* reared on Diet 1 (replacement diet), Diet 2 (Khan’s diet for Anopheles), Diet 3 (Khan’s modified diet) and Diet 4 (IAEA diet) were analyzed using the age-stage, two-sex life table software. Diet 4 (IAEA) was used as a control for comparison. The results indicated that significantly maximum percentage of egg hatching of *Ae. aegypti* was observed when reared on Diet 4 (73.86%) and Diet 3 (72.90%), while less % of egg hatching was recorded in Diet 1 (40.67%) and Diet 2 (55.53%). The data further showed that the Diet 3 had a highest intrinsic rate of increase (*r)* (0.097 ± 5.68 day^−1^), finite rate of increase (*λ*) (1.10 ± 6.26 day^−1^) and net reproductive rate (*R*_*0*_) (11.99 ± 1.52 eggs/female) followed by Diet 2 and Diet 4. The mean generation time (*T*) of *Ae. aegypti* reared on Diet 3 (23.67 ± 0.86 days) and Diet 1 (24.05 ± 0.61 days) was significantly shorter than Diet 2 (26.15 ± 0.71 days) and Diet 4 (26.41 ± 0.38 days). The overall results revealed that Diet 3 showed good results at different life table parameters of *Ae. aegypti* and can be used as the preferred diet in the Sterile Insect Technique (SIT) where the mass culture of mosquitoes is required.

## Introduction

Mosquitoes belong to the family Culicidae, which are a medically and commercially important group of dipteran insects that act as vectors for a variety of human and zoonotic diseases^1,2^. They are a large category of partially aquatic arthropods. These arthropods transfer a variety of pathogens that cause serious illnesses such human malaria, dengue fever, filariasis, and viral encephalitis, in addition to other ailments^3^. More than 3600 mosquito species exist worldwide, divided into 39 genera and 135 subgenera^4^. Dengue fever is a severe concern worldwide among mosquito-borne diseases^5^. The dengue virus (DENV) causes an acute infectious disease that is spread by *Aedes* mosquitoes. It is the world's fastest growing arboviral disease, with outbreaks dating back to 1779^6^. Dengue fever has moved to new geographic regions and contexts (e.g., from urban to rural areas) throughout Asia, Africa, North, and Latin America, with an estimated 30-fold increase in incidence during the last 50 years. Every year, over 400 million people are predicted to fall unwell^6^. The traditional pest management techniques have so far been unable to maintain this species at levels that are safe and sustainable^7^. The indiscriminate use of broad-spectrum pesticides may cause major problems with overdosing of pesticides and pest resistance, killing of beneficial insects and environmental contamination. The sterile insect technique (SIT), applied as part of an area-wide integrated pest management approach (AW-IPM), offers considerable potential and has been used with great success against major pests of agricultural importance for eradication, suppression, or establishing pest-free areas. Under the joint program of FAO and WHO, the sterile insect technique has received much impetus all over the word for the suppression of vectors of deadly diseases. Under the SIT program, the males used must be compatible with the wild males in vigor, flight and selection of mate. Much of this potential is determined by the diet provided in the larval stages^8^. For laboratory rearing, artificial diets have been developed by mixing products from livestock, cereals, yeast and algae^9^. A life table is a basic population model that may be used to examine the dynamics of a species' population, including life demography and general biology, which covers a population's survival, growth, and reproductive system under various conditions^10,11^. Several factors influence mosquito survival, fecundity and mortality^12^.

A few scientists have studied some demographic parameters of selected strains of the *Ae. aegypti* under laboratory conditions. Temperature, rainfall, the type and quantity of larval aquatic habitats, insecticidal applications and proximity to human habitations are all important ecological factors^13,14^. The quality of released males with the right phenotypic quality can be improved by changing the conditions of larval rearing. For healthy adult life histories, proper nutrient intake during the larval stage is crucial as the larvae consume more food for their growth^15^.

The Sterile Insect Technique (SIT) is a biological strategy to insect pest management that entails releasing a large number of mass-reared and sexually sterile males over a lengthy period of time to reduce the fertility of a population of the same species. Effective control can be achieved if sterile insects are utilized frequently as part of integrated pest management for the entire affected region^16,17^.

The SIT is required for both the colonization of the target species and the extensive breeding of competitive and healthy males capable of locating and mating with wild females. The mass rearing and colonization processes must therefore maintain and, if at all feasible, enhance the traits necessary for these roles^18^. The component selection should be taken into account when choosing a diet for mass rearing as the quality of the larval food and rearing conditions have a direct and frequently irreversible effect on adult attributes^15,19^. Diets containing ingredients like wheat germ, soyflour, ground beef, and chicken eggs are relatively inexpensive and many are highly nutritious, however, rearing many successive generations of fecund insects on a chemically dependent diet would be more expensive and logistically challenging. Such diets would also be unaffordable both in terms of the price per kilogram and the amount of preparation time. The larval diet should include a variety of nutrients in order to lower the risk of nutritional inadequacies that could have a negative impact on both the rearing output and the fitness of the males produced^20^.

The FAO/IAEA diet, which was initially employed to mass-produce healthy male Anopheles mosquitoes for conventional SIT applications, was modified as IAEA 2 diet containing necessary fatty acids and amino acids required for mosquitoes development^21,22^. However, in terms of cost, a low-cost composite larval diet, readily available for Anopheles economic rearing that included beans, corn, wheat, chickpea, rice, and beef liver (BCWPRL)^9^. Numerous nutrients, including carbohydrates, amino acids, polyunsaturated fatty acids (PUFAs), unsaturated fatty acids, vitamins and minerals were reported to be present in this diet as high energy reserves for male longevity^21^. In terms of cost, the plant derived ingredients are much lower than that of bovine liver and therefore, it was hypothesized that addition of plant derived ingredients will not affect the diet quality but reduces the price per kg of the final diet. The common auster mushroom (*Agaricus bisporus*) has been reported as excellent source of protein with 19–38% of protein on a dry weight (DW) basis^23,24^. The present study was conducted to examine the performance of several known larval diets including a replacement diet in which the bovine liver (BL) was replaced with the auster mushroom powder as a replacement of animal protein to plant protein and another modified diet in which the ratio of the ingredients was changed for Aedes rearing to understand its effect on life table parameters of *Ae. Aegypti* rearing.

## Methods and materials

The study regarding the effect of different diets on the life table parameters of *Aedes aegypti* was carried out in the medical entomology laboratory at NIFA, Peshawar-Pakistan during 2022.

### Mosquito rearing and laboratory colony maintenance

For eggs hatching and rearing mosquitoes, one liter of water was boiled for 2–3 min and then poured into a glass jar covered with a lid at the top. After its cooling to approx. 40 °C, fresh eggs card of *Ae. aegypti* were added into the jar along with 100 μl of the IAEA diet for larval feeding. Led of jars were closed and left for incubation for next 12–15 h. Larvae after hatching along with jars content were transferred from the jar into the enameled larval trays containing 1.5 L of tap water. Each tray was containing approximately 150–200 larvae. The developing larvae were provided daily 1 to 2 ml of 3% of the IAEA diet. The trays were checked daily for pupation. After pupal development, they were collected from enameled trays into the cups. Cups containing pupa were placed into the clean cage. The laboratory temperature was kept at a constant temperature of 27 ± 2 °C with a relative humidity of 65 ± 5 percent. After pupation, all pupae were collected with help of plastic dropper in small plastic cup and put inside Bug Dorm adult cage (30 × 30 × 30cm) for adult emergence. The male female ratio was maintained at 1:1 in all cages to mate freely. Cleanness of cages and fresh sugar solution was prepared once every week. Blood feeding of female mosquitoes was done with de-fabricated bovine blood for 30 min through para film membrane for three consecutive days. Plastic ovi-cups lined with filter papers were prepared for female’s egg laying. Whatman filter paper (7 × 2inch) was wrapped inside the ovi-cups for egg laying and ⁓10 ml tape water was added into each cup to maintain wetness of the filter paper. Cups were placed every Friday afternoon in the rearing cages and removed on Monday. The filter papers with deposited eggs were dried at room temperature for future use and colony maintenance.

### Diet experiments

Experiments were conducted to evaluate the effects of various larval diets on life table parameters of *Ae. aegypti* with the following composition (Table 1).

**Table 1:** List of diets along with composition to be used for studying life table parameters of *A. aegypti.*

Diets	Composition ingredients (in grams)
Diet 1 (Replacement diet)	(Chickpea + beans + rice + mung beans and auster mushroom powder) (2 + 2 + 2 + 2 + 2) g
Diet 2 (Khan’s diet for Anopheles)	(Bean + corn + wheat + chickpea + rice + bovine liver powder) (2 + 2 + 2 + 2 + 2 + 2) g
Diet 3 (Khan’s modified diet)	(Bean + corn + wheat + chickpea + rice + bovine liver powder) (2 + 3 + 2 + 3 + 3 + 3.6) g
Diet 4 ( (IAEA diet)	(brewer’s yeast + bovine liver powder + tuna fish meal powder) (3.5 + 9 + 12.5) g

*Diet 3 is the modified diet formulation of diet 1 developed by Khan, (Khan unpublished data) for Aedes laboratory colony maintenance.

### Procedure for diets preparation

All the ingredients used in the experiments were washed with clean water and then sun-dried. After drying the different ingredients were ground in mortar and pestle to powder form. The different ingredients of each diet were mixed and put in separate jars. The slurry of all diets at 3% concentration was prepared by dissolving 3 g of each diet mixture diluted in 97 ml of distilled water. After preparation, all diets were refrigerated at 4 °C and replaced with new preparation once every week.

### *Aedes aegypti* life table study

*Aedes aegypti* life table study was conducted using four different diets. Eggs cards containing sufficient number of *Ae. aegypti* eggs were used from general colony. These eggs were counted on filter paper under a stereo microscope. The filter paper containing ⁓ 300 eggs were hatched initially in one-liter pre-boiled water at approx. 40 °C. Fresh eggs card of *Ae. aegypti* were added into the jar along with 640 μl of respective diets. All jars were labeled properly. Fifty newly hatched 1^st^ instar larvae from each jar were collected per replicate and added into 14 cm glass Petri dishes containing 50 ml distilled water providing density level of 1 larvae/ ml water. An aliquot of 640 µl of respective diet prepared at 3% concentration was added daily at 8:30 am into each petri dish for the first two days and there after twice a day; first meal at 8:30 am and 2nd at 4:0 pm till pupa formation. All petri dishes containing larvae in respective diets were placed in the medical entomology laboratory maintained at 27–28 °C and 50–60% relative humidity. Daily observation on larval development, mortality and pupa formation was recorded. Upon pupa formation, they were counted and collected in emergence cups and placed in adult cages labelled with diet names. After emergence, the sex ratio in each cage was maintained at 1:1 by adding or removing males from one replicate cage to another with the help of a mouth aspirator. All diets were replicated three times and adults (both male and females) were fed with 10% sugar solution from sugar feeders as usual.

The females were blood fed for three consecutive days (Wednesday through Friday) in a week with live albino mice. Permission for use of mice was obtained from the ethical committee of NIFA. In all cages, the mortality and number of males and females were checked daily. Ovi-cup lined with wet filter paper was placed in adult cages for eggs laying and egg cards with newly laid eggs were collected every Monday. The eggs hatch data was recorded from eggs produced by the parents which were reared using different larval diets. The data was recorded continuously till all individual died in each treatment.

The *Aedes egypti* survival rate (*s*_*xj*_) for each age-stage of was computed as ${s}_{xj}=\frac{{n}_{xj}}{{n}_{01}}$, where “*n*_*01*_” (the number of eggs used at the beginning of life table study) and “*n*_*xj*_” (the number of insects that survived to age *x* and stage *j*).

The age-specific survival rate (*l*_*x*_) of *Ae. egypti* was determined by the formula^25^, ${l}_{x}={\sum }_{j=1}^{m}{s}_{xj}$. The female age-specific fecundity “*f*_*x4*_*”* of *Ae. egypti* reared in groups was estimated by the formula^26^ as, ${f}_{x4}=\frac{{E}_{x}}{{n}_{x4}}$ , Where (*E*_*x*_) is the total number of eggs laid by all female adults together (the 4^th^ life stage) at age x, female age-specific fecundity “*f*_*x4*_” .

The net reproductive rate (*R*_*0*_) is defined as the average number of offspring that a female produces during her lifetime is called the net reproductive rate (*R*_*0*_). The net reproductive rate which takes into account the survival rate, is the total of all *l*_*x*_*m*_*x*_ (age-specific maternity) was calculated as follows^25^; ${R}_{0}=\sum_{x=0}^{\infty }\sum_{j=1}^{k}{s}_{xj}{f}_{xj}$, Where “*k*” is the number of life stages. The Lotka–Euler equation was used to compute the intrinsic rate of rise (*r*) with age indexed from zero and is defined as a measure of the maximum potential growth rate of a population in the absence of any limiting factors such as resource availability or predation and is calculated as^27^,

$$\mathop \sum \limits_{x = 0}^{\infty } e^{{ - r\left( {x + 1} \right)}} l_{x} m_{x} = 1$$

Similarly, the Grass reproductive rate (*GRR*) of *Ae. egypti* was estimated using equation as $GRR=\sum {m}_{x}$ .The finite rate of increase (λ) of *Ae. egypti* was calculated as $\lambda ={e}^{r}$.

The group-rearing method's age-specific life expectancy (*e*_*x*_*;* i.e. how long individuals of age x are expected to survive) of *Ae. egypti* was estimated as follows^28^:

$$e_{x} = \frac{{\mathop \sum \nolimits_{i = x}^{n} l_{i} }}{{l_{x} }}$$

*l*_*i*_ = the probability that an individual of age *0* will survive to age *i.*

According to the age stage, two-sex life table theory, the reproductive value (*v*_*x*_) of *Ae. egypti* was calculated^29,30^ as:

$$v_{x} = \frac{{e^{{ - r\left( {x + 1} \right)}} }}{{l_{x} }}\mathop \sum \limits_{i = x}^{\infty } e^{{ - r\left( {i + 1} \right)}} l_{i} m_{i}$$

As the *Ae. egypti* populations were reared on different diets in groups, the group-reared data was first transformed into individual-reared data in order to estimate standard errors of the various life-table parameters. The paired bootstrap method was applied with 100,000 resamples to analyses differences between treatments^31,32^.

The different parameters i.e. survival rate (*s*_*xj*_), age-specific survival rate (*l*_*x*_), age specific fecundity (*m*_*x*_), net reproductive rate (*R*_*0*_)_,_ intrinsic rate of increase (*r*), Grass reproductive rate (*GRR)*, finite rate of increase (λ), age-specific life expectancy (*e*_*x*_) and reproductive value (*v*_*x*_) were analyzed and compared using TWOSEX-MSChart computer program^33^. The data regarding the percentage eggs hatch rate were subjected to one way analysis of variances using Statistical package (Statistix 8.1) and means were compared using LSD test at 5% level of probability.

### Ethical approval

All the experiments were reviewed and approved by the Department of Plant Protection, The University of Agriculture, Peshawar and Nuclear Institute for Food and Agriculture (NIFA) ethical committees as per set guidelines and were in accordance with the Pakistan Agricultural Pesticide Act. 1997.

## Results

Different larval diets were used in rearing of *Ae. aegypti* under laboratory conditions at NIFA, Peshawar during 2022. The effect of larval diets against the different stages of *Ae. aegypti* were calculated. The results of the experiment are given below.

### Effect of different larval diets on mean percent eggs hatching of *Aedes aegypti*

The data in Table 2 shows that variable number of mean percent eggs hatching of *Ae. aegypti* were observed when reared on different diets. The data further shows significantly highest mean percent egg hatching (72.90%) was recorded for diet 3, this was followed by diet 4 (37.86%) as compared to diet 1 and diet 2 with 40.66% and 55.53% eggs hatching respectively.

**Table 2:** Overall eggs hatched data Percentage of *Aedes aegypti* reared on different diets under laboratory conditions.

Diets	Egg Hatching (Mean %)
Diet 1 (Replacement diet)	40.667c
Diet 2 (Khan’s diet for Anopheles)	55.533b
Diet 3 (Khan’s modified diet)	72.900a
Diet 4 (IAEA diet)	73.867a
LSD value	11.510

Mean followed by different letters in columns are significantly different at α = 0.05 using LSD test.

### Population parameters of *Aedes aegypti* reared on different larval diets

The data regarding the different population parameters of *Aedes* is shown in Table 3. The bootstrap approach was used to compute the average and standard error. The data shows that *Ae. aegypti* reread on diet 3 showed significantly maximum intrinsic rate (*r*) and finite rate of increase (*λ*) (0.097 ± 5.68 per day, 1.102 ± 6.26 /day offspring) (*P* = 0.7032; 0.7028) as compared to mosquitoes reared on diet 1, diet 2 and diet 4. The highest net reproductive rate (*Ro*) (11.99 ± 1.52 offspring) of *Ae. aegypti* was also observed for diet 3 followed by diet 1 (10.53 ± 1.60 offspring), diet 2 (9.07 ± 136 offspring) and diet 4 (5.75 ± 072 offspring) respectively., while significant difference was recorded in the net reproductive rate of *Ae. aegypti* reread on diet 3 and diet 4 (*P* = 00,028).

**Table 3:** Effects of different larval diets on the life table parameters of *Aedes aegypti* reared in groups.

Treatment	n*	The intrinsic rate of increase (*r)* per day (Mean ± S.E)	The finite rate of increase (*λ*) per day (Mean ± S.E)	The net reproductive rate (*R*_*0*_) offspring (Mean ± S.E)	The mean generation time (*T*) in day (Mean ± S.E)	Gross reproductive rate (*GRR*) offspring (Mean ± S.E)	Doubling Time (*DT*) (Mean ± S.E)
Diet 1 (Replacement diet)	300	0.094 ± 0.00483a	1.099 ± 0.0053a	10.53 ± 1.60a	24.05 ± 0.71b	57.41 ± 4.62b	10.46 ± 0.74a
Diet 2 (Khan’s diet for Anopheles)	240	0.093 ± 0.00718a	1.097 ± 0.0078a	9.07 ± 1.36a	26.15 ± 0.38a	56.21 ± 4.38c	7.43 ± 0.60b
Diet 3 (Khan’s modified diet)	250	0.097 ± 0.00568a	1.102 ± 0.0062a	11.99 ± 1.52a	23.67 ± 0.86b	112.49 ± 16.09a	7.08 ± 0.42d
Diet 4 ( IAEA diet)	230	0.066 ± 0.00450b	1.068 ± 0.0048b	5.75 ± 0.72b	26.41 ± 0.61a	29.24 ± 2.38d	7.29 ± 0.38c

n* no of individuals at the start of the experiment.

Mean followed by different letters in columns are significantly different using bootstrap method.

Table 3 further displayed that the mean generation time (*T*) of *Ae. aegypti* reared at larval stages on diet 2 and diet 4 was significantly different from mosquitoes reared on diet 3 (23.67 ± 0.86 days) and diet 1(24.05 ± 0.71 days). The significantly highest gross reproductive rat (*GGR*) of *Ae. aegypti* was recorded when reared on diet 3 (112.49 ± 16.9), followed by diet 1 (57.41 ± 4.62), diet 2 (56.21 ± 4.38) respectively, while significantly minimum *GRR* value was obtained in diet 4 (29.24 ± 2.38) (*P* = 0.00). The doubling time (*DT*) of *Ae. aegypti* reread on diet 1 was significantly higher (10.46 ± 0.74 days) as compared to diet 2 and diet 4 (7.43 ± 0.60, 7.29 ± 0.38 days) respectively, while significantly lowest *DT* was observed from diet 3 (7.08 ± 0.42 days) (*P* = 0.00103).

**Figure 1:**
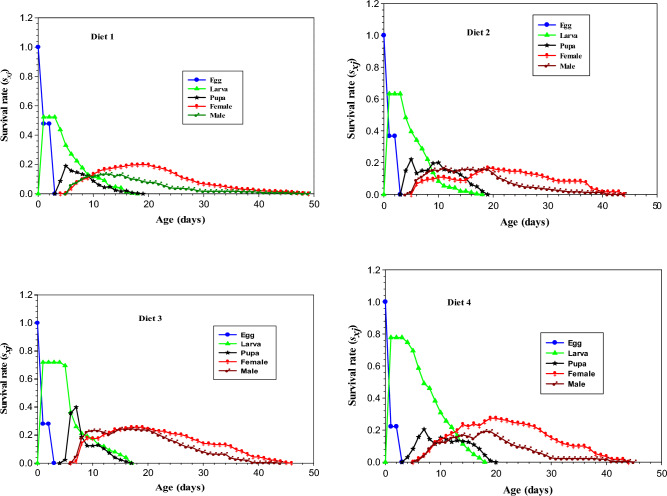
Age-specific Survival rate (*s*_*xj*_) of *Aedes aegypti* reared on different larval diets under laboratory conditions.

The curves in Fig. 1 shows that a higher survival rate (*s*_*xj*_) during the egg stage of *Ae. aegypti* was observed when reared on diet 1 (47%), this was followed by diet 2 and diet 3 with 36% and 28% respectively. The lowest egg survival rate (*s*_*xj*_) was recorded when reared on diet 4 (22%). Furthermore, the curved line related to the larval stage of *Ae. aegypti* reared on different diets showed that the survival rate was in the range of (63%—72%) when reared on diet 2 and diet 3. The curve in Fig. 1 further shows that the maximum survival rate (*s*_*xj*_) was recorded when mosquitoes at the larval stage were reared on diet 4 (77%), while the minimum survival rate (*s*_*xj*_) of larvae was observed when reared on diet 1 (52%). The curve regarding the survival rate (*s*_*xj*_) of the pupal stage of *Ae. aegypti* when reared on different diets shows that the higher survival rate (*s*_*xj*_) was recorded in diet 3(35%), followed by diet 2 and diet 4 with 22% and 20% respectively. The lowest survival rate (*s*_*xj*_) was recorded in diet 1 (15%) (Fig. 1). Furthermore, the curves regarding the survival rate (*s*_*xj*_) of the adult stage of *Ae. aegypti* when reared on different diets shows that the higher survival rate (*s*_*xj*_) of female mosquitos was recorded in diet 4 (0.27%), followed by diet 1 and diet 3 with 19% and 25% survivorship respectively. While the lowest survival rate (*s*_*xj*_) of female mosquitoes was observed when reared at larval stage on diet 2 (16%). The male mosquitos of *Ae. aegypti* reared on different diets show that the survival rate (*s*_*xj*_) was in the range of 13–24%. The curve further shows that the maximum survival rate (*s*_*xj*_) of a male adult was noted when reared on diet 3 (24%), while the minimum survival rate (*s*_*xj*_) was recorded when reared on diet 1(13%).

**Figure 2:**
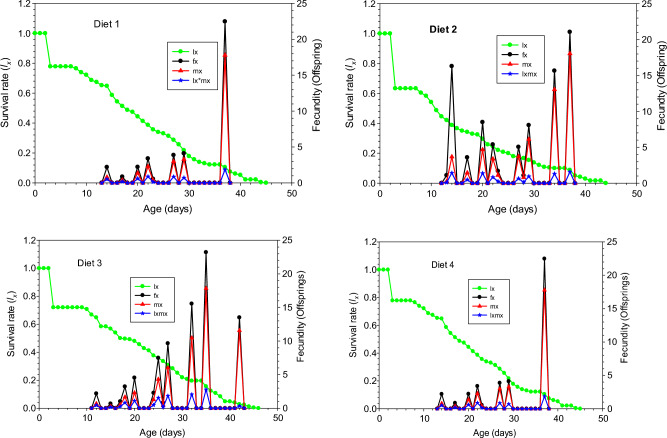
Age-specific survival rate (*l*_*xj*_), age-stage fecundity of the female stage of (f_x_), age-specific fecundity (*m*_x_), and age-stage maternity (*l*_*x*_*m*_*x*_) of *Aedes aegypti* reared on different diets under laboratory conditions.

The age-specific survival rate (*l*_*x*_) of *Ae. aegypti* curve in Fig. 2 dropped quickly on all diets on day 3. In addition, the first age at which the survival rate dropped to 0.00 occurred at age 46 days on Diets 3 which was one day later than those reared on the diet 1 and diet 4 (45 days) and two days later than Diet 2 which was 44 days. The highest age-specific fecundity *(fx)* (23.47 offspring day^−1^) was noted on day 36 in Diet 3 followed by Diet 1 (22.47 offspring day^−1^) on day 40 and Diet 2 (21.05 offspring day^−1^) on day 38. The curve of the age-specific fecundity (*m*_*x*_) revealed that the egg laying of *Ae. aegypti* started in Diet 3 on day 13 and maximum egg laying (17.84 eggs per day) was recorded on day 36.

The highest age-specific maternity (*l*_*x*_*m*_*x*_) of *Ae. aegypti* (2.78 offspring) was observed on Diet 3 followed by Diet 1 with (1.85 offspring) and Diet 2 with (1.57 offspring) while lowest offspring of 1.85 offspring was recording when reared on Diet 4.

### Age-specific life expectancy of *Aedes aegypti* on different diets

**Figure 3:**
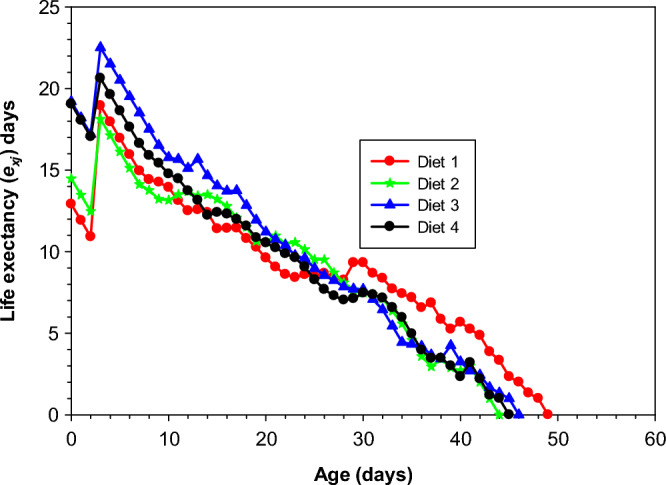
Age-stage specific lifetime probability (*e*_*xj*_) of *Aedes aegypti* raised in a lab environment on various diets.

The different curves in Fig. 3 revealed that the life expectancy of newly laid eggs of *Ae. Aegypti* was shortest when reared on Diet 2 (18.13 days) on day 3 and longest in Diet 3 (22.51 days) on day 3. While the life expectancy of *Ae. aegypti* reared on Diet 1 and Diet 4 were (18.95 days) and (20.63) days respectively.

### Age-specific reproductive value of *Aedes aegypti* reared on different diets

**Figure 4:**
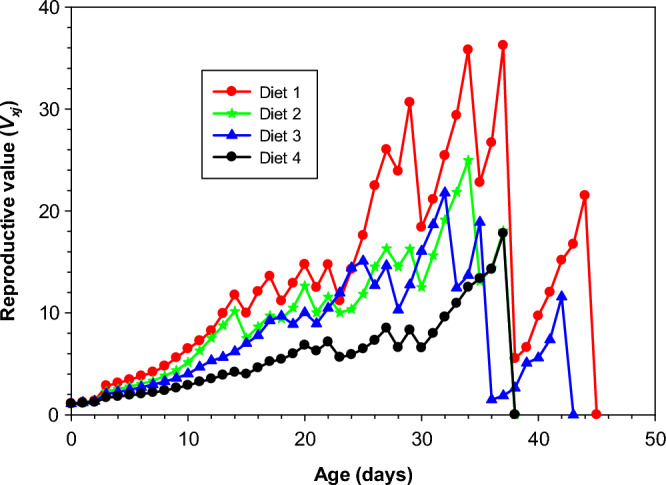
Age-stage specific reproductive valve (*v*_*xj*_) of *Aedes aegypti* reared on different diets under laboratory conditions.

The curves in Fig. 4 revealed that the different diets significantly affected the reproductive value of the *Ae. aegypti*. Peak reproductive value (v_*xj*_) of (36.23offspring per day) was recorded when reared on Diet 1 on day37. While the lowest reproductive value of *Ae. aegypti* was observed in Diet 4 (17.79 at age 37 day). In others diets like Diet 2 and Diet 3 peak reproductive values were 24.98 and 21.79 offspring per day at day 34 day and day32 respectively.

## Discussion

Optimization of mass production of *Ae. aegypti* requires efficient rearing system and provision of diets in the early stages of development for production of healthy individuals. Much of this potential is determined by the diets provided in the early stage of rearing^34^.On the other hand, adult quality assessment is based on intrinsic rate of increase (*r*), Grass reproductive rate (*GRR*), finite rate of increase (*λ*), net reproductive rate (*R*_*0*_) for its mass rearing, and flight ability, mating competitiveness with wild mates^35^. At the end when diet provides all the key parameters of high quality, the next critical question is whether the diet components are easily available and not so much expensive^36,37^.

Keeping in view, all above requirements for an acceptable assessment of diets, we evaluated four larval diets; one developed by IAEA for Aedes rearing (IAEA diet), another diet developed for Anopheles rearing (Khan’s diet), one replacement low-cost diet in which the costly animal protein (liver powder) in which the animal protein was replaced with plant protein and another modified diet developed for Aedes rearing. These diets were specifically chosen to be used for mass rearing, with the aim of providing adequate nutritional components to optimize larval rearing and enhance adult quality. The ingredients of some of comparing diets were chosen for their worldwide availability, comparatively low cost and high nutrient quality^38,39^. We expected higher or equal larval survivorship, rapid development and survivorship of adults from replacement diet. The diet 3 was modified to enhance energy reserves for male Aedes when sterilized and used in a sterile insect technique program for competing with wild males^40^. It has been reported earlier that at least 14 amino acids, sugars, polyunsaturated fatty acids (PUFAs), sterols, and nucleotides are needed for successful rearing^41^ and a minimum number of essential vitamins are required for optimum growth, larval development, survival, and adult flight in mosquitoes^42^. A major amount of these ingredients is present in the Khan’s and the IAEA diets^9^. However, in terms of cost, the bovine liver powder (BL) is the only costly ingredient in Khan’s diet that was replaced with common auster mushroom powder (a replacement diet) and investigate its effect on *Ae. aegypti* rearing using life table parameters under age-stage two-sex life table theory.

The results indicated that significantly the highest percentage of egg hatching was observed from both IAEA (73.867%) and Khan’s modified diet (72.90%), while the lowest % of egg hatching was recorded from the other two diets. Diet 3 (Khan’s modified diet) also resulted in shorter larval period, early pupation and adult emergence as compared to other three diets. Other researchers also reported longer larval development time of mosquitoes reared on IAEA recommended diet^37^. Similar results were also obtained by other researchers who stated that larvae reared on IAEA recommended diet have longer developmental time, longer life span but show high endurance against rising temperature as compared to other tested diets^43^. No appreciable variation in fecundity was reported by these authors except high larval survival and adult growth was observed when mosquitoes were fed on diets containing tuna meal and bovine liver powder.

Several other researchers reported that if the value of “*R”* is greater than 1, the population of the species increases. They further reported that when the value of “*R”* is equal to 1, the population of the species remains stable and when this value is less than 1, the population declines and the species goes extinct^44,45^. When the quantity of some diet ingredients in Diet 2 was increased (Diet 3) and fed it to larvae of *Ae. aegypti* at 3% concentration, the larval developmental parameters improved. Prominent increase in the intrinsic rate of increase (*r)* (0.097 ± 0.0056 day^−1^), finite rate of increase (*λ*) (1.10 ± 0.0062 day^−1^) and net reproductive rate (*R*_*0*_) (11.99 ± 1.52 eggs/female) was recorded from diet 3. The intrinsic rate of increase, finite rate of increase, and net reproductive rate are not typically used to measure the quality of food. Instead, these parameters are used in the growth and reproductive potential estimation of insect pests^48^. Because the parameters *r* and *λ* depend upon the fecundity and growth of individuals, therefore differences in these parameters may affect the expansion rates of populations^49,50^. These results are further in line with other researcher’s findings who have documented significant variations in demographic life table parameters of pests including *Sitotroga cerealella*, *Plutella xylostella, Melanoplus femurrubrum* and *Parapoynx crisonalis*^51–54^.

The mean generation time (T) of *Ae. aegypti* reared on Diet 3 (23.67 ± 0.86 day) was also significantly lower than Diet1 (24.05 ± 0.71 days) and Diet 2 ((26.15 ± 0.38 days) respectively. Undernutrition may extend the life span of certain animal species including *Ae. Aegypti.* Adult males of the *Culex quinquefasciatus* species lived longer when they were fed a low-food larval diet, whereas females survived longer when they were fed a high-food larval diet^46,47^. Contrary to our expectations, the replacement diet could not provide comparable results. This might have been due to the less amount or quality of protein content present in the plant base mung beans and auster mushroom. Further studies may investigate proximate composition of protein and mineral contents in each diet ingredient or its composition in mixture diets and decide on increasing the amount of mung bean and auster powder in the replacement diets.

## Conclusion

The current study concluded that different diets significantly affected the growth and survival rates of *Ae. aegypti*. However, the Diet 3 provided high grass and net reproductive rate, and finite rate of increase of *Ae. aegypti* and therefore, could prove valuable for mass culturing of *Ae. aegypti* in an SIT program for managing dengue disease.

## Data Availability

The datasets used and/or analyzed during the current study are available from the corresponding author on reasonable request.
